# Alterations in physiological and biochemical characteristics of *Prunus sibirica* seedlings raised from spaceflight seeds

**DOI:** 10.1371/journal.pone.0321147

**Published:** 2025-04-24

**Authors:** Ying Kang, Yuncheng Zhang, Jianhua Chen, Qingfu Yu, Biao Li, Yijin Wang, Shengjun Dong

**Affiliations:** 1 Key Laboratory of Tree Genetics, Breeding and Cultivation in Liaoning Province, Shenyang, Liaoning, China,; 2 College of Forestry, Shenyang Agricultural University, Shenyang, Liaoning, China,; 3 Liaoning Kazuo County Forestry Seedling Management Station, Chaoyang, Liaoning, China; Institute for Biological Research, University of Belgrade, SERBIA

## Abstract

The aim was to explore the alterations in growth traits, physiological and biochemical characteristics of *Prunus sibirica* seedlings raised from spaceflight seeds. The seedlings cultivated by the “Shenzhou XII” spacecraft carrying the seeds of superior clones of *P. sibirica* were used to observe their growth traits and determine physiological indicators. The results showed that plant height of *Prunus sibirica* seedlings raised from spaceflight seeds increased by 18–34% and internode length increased by 8–26%, but the number of primary branches, secondary branches, and leaves showed no significant change compared to the ground control. Leaf length and width of *Prunus sibirica* seedlings raised from spaceflight seeds were significantly higher than those of the ground control, with leaf length, width, and area increasing to 1.21–1.80 times higher than that of the ground control. Furthermore, the antioxidant and osmoregulatory capacities of *P. sibirica* seedlings raised from spaceflight seeds were altered. The peroxidase (POD) activity and Malondialdehyde (MDA) content were increased in ST28, ST207, and ST507, while they were reduced in ST1 and ST453. Compared with the ground control, the content of soluble sugar(SS), starch (St), and free proline (Pro) were significantly or highly significantly increased in all lines. The content of soluble protein (SP) was significantly increased in ST1, ST28, ST207, and ST507, while there was no significant change in ST453. *P. sibirica* seedlings raised from spaceflight seeds exhibited increased leaf pigment content, the interstitial CO_2_ concentration (Ci), net photosynthetic rate (Pn), stomatal conductance (Gs), transpiration rate (Tr). In conclusion, compared with the ground control, the growth indexes and physiological characteristics of *Prunus sibirica* seedlings raised from spaceflight seeds were changed, and the direction of change was different for different lines. This provided a foundation for subsequent germplasm improvement and variety selection.

## Introduction

*Prunus sibirica* is a plant of the genus *Prunus* in the Rosaceae family, which is resistant to cold and drought, adapts to barren environments, and has a high ecological, economic, and social value [1]. *P. sibirica* has a wide range of applications and developmental potential in various industries [2,3]. Many nutritional and medicinal components are found in kernels of *P. sibirica*, which are widely used in food, industry, and medicine. The total cultivation area of *P. sibirica* in China is approximately 2 million hm^2^. The annual production of *P. sibirica* kernels is approximately one million tons, and approximately 40,000 tons of almonds are exported [4]. With the increasing demand for kernels and almonds of *P. sibirica* in both the domestic and international markets, the yield and quality requirements of *P. sibirica* have also increased. However, owing to poor field management, the long-term natural reproduction of *P. sibirica* has resulted in a decline in population traits, yield, quality, and resistance [5]. The market for *P. sibirica* has seen demand for outstrip supply. *P. sibirica* is currently mainly bred using conventional breeding methods, such as selective breeding and hybridization, and has a long breeding cycle and low mutation frequency, which severely restricts the development of the *P. sibirica* industry [6]. Therefore, there is an urgent need to adopt other rapid and efficient breeding methods to improve the speed of variety selection and accelerate seeding.

Spaceflight treatment is the process of transporting plant seeds, branches, bulbs, and other materials into space through spacecraft or return satellites. It utilizes the special environment of space that is different from the ground, including space radiation, microgravity, and high vacuum, to change the materials carried on board. After returning to the ground, it selects materials with excellent properties for subsequent research [7,8]. Compared with conventional breeding methods, spaceflight treatment can rapidly obtain a large amount of high-quality material, providing a good basis for breeding new high-quality germplasms [9]. Plants have been transported into space since 1987, and more than 1,000 plant seeds and biological materials have been subjected to spaceflight treatment experiments in China. Spaceflight has been widely used to breed crops, vegetables, and medicinal plants; however, its application in woody plant is relatively limited [10]. To date, successful results using return satellites and spacecraft to carry seeds of forest trees have been observed in *Populus ussuriensis* [11], *Betula pendula* [12], *Robinia pseudoacacia* [13], *Pinus armandii*, *Xanthoceras sorbifolia*, and *Acer mono* [14]. However, few follow-up studies have been conducted on the ground cultures of spaceflight-treated woody plant materials.

Currently, long-term manned space stations have been established in China; therefore, spaceflight is an effective method to study space biology [15]. The space environment is superior to the ground and contains many unique features, such as special magnetic fields, microgravity, cosmic radiation, and ultrahigh temperatures [16–20], which may cause changes at the morphological, physiological, molecular, and metabolic levels [21,22]. After spaceflight treatment, the plant height and ground diameter of *Avena sativa* [23] and *Acer mono* [14] were higher than those of the ground control. The leaf shape of *Cinnamomum cassia* changed from long elliptical to elliptical, the leaf tip became obtuse, and leaf length, width, and area significantly increased [23]. Spaceflight treatment promoted seed viability in four lines of *Betula platyphylla*, resulting in dwarfing and reduced chlorophyll (Chl) content in 1-year-old seedlings [24]. The antioxidant enzyme activities of POD and superoxide dismutase (SOD) in *Acer mono* leaves were significantly higher than those in the ground control after spaceflight treatment. Using RAMP and SSR molecular marker technology, heritable variations have been identified in the genome of *Acer mono* after spaceflight treatment at the molecular level [14,25]. All photosynthetic indices of the third generation of *Salvia miltiorrhiza* showed significant differences compared to the ground control [26].

In this study, we took the seedlings cultivated from high-quality *P. sibirica* clone seeds carried by the “Shenzhou XII” spacecraft as the test material (ST), and the seedlings cultivated from the seeds that had not been spaceflight treated as the ground control (GC). The activities of antioxidant enzymes (SOD, POD, catalase (CAT)), osmoregulatory substances (soluble sugar (SS), starch (St), free proline (Pro), and soluble protein (SP)), photosynthetic pigments (Chl a, Chl b, Chl, Carotenoids (Car)), and photosynthetic properties (the interstitial CO_2_ concentration (Ci), net photosynthetic rate (Pn), stomatal conductance (Gs), transpiration rate (Tr)) in *P. sibirica* seedlings after spaceflight treatment were investigated. Furthermore, the growth traits and leaf morphology of seedlings were observed in the field to reveal the alterations of *Prunus sibirica* seedlings raised from spaceflight seeds and to provide a theoretical basis and new ways for *P. sibirica* breeding in the future.

## Materials and methods

### Plant materials and treatment

Test materials were obtained from the Shenyang Agricultural University National Forest Germplasm Resource Conservation Bank of *P. sibirica*. Five productive *P. sibirica* clones were selected from the following seed sources: No.1 (Kazuo, Liaoning, China), No.28 (Aohan Banner, Inner Mongolia, China), No.207 (Huining, Gansu, China), No.453 (Zalantun, Inner Mongolia, China), and No.507 (Zabaykalsky Krai, Russia). The seeds of the same line were divided into two groups, one for spaceflight treated (ST) aboard the Shenzhou XII spacecraft from June 17, 2021, which returned to the ground on September 17, 2021, after 90 d of operation, and the other for ground control (GC), which were stored at 25°C in a dry environment. On November 1, 2021, the ST and GC seeds were soaked in cold water for 7 days and then sown in a nutrient bowl, with one seed in each pot, covered with 2–3 cm of soil after sowing, and watered thoroughly. The experiment was conducted in a randomized complete block design with seven blocks, with each block containing one plant from each of the five lines of spaceflight treatment and ground control placed at a spacing of 50 × 50 cm. Seedling emergence began after thawing water was poured at the beginning of April 2022, when conventional field management was carried out, and the seedling emergence rate reached 100% for all lines after 30 d.

### Determination of growth traits

Growth traits were measured in September 2022, after cessation of growth in *P. sibirica*. Plant height was measured using steel tape, ground diameter and internode length were measured using a digital caliper, and internode length was measured eight times per plant. Mature leaves from the middle of branches in the east, south, west, and north directions were selected for each seedling. The length and width of the leaves, length and thickness of the petiole, and length of the leaf tip were measured using digital calipers. The leaf area was measured using a leaf area meter, and the eight leaves were collected from each seedlings with uniform growth were selected.

### Sample collection

In mid-July 2022, leaves were collected from three seedlings with uniform growth were selected from each line of ST and GC, and mature leaves were collected from the middle of the branches of each seedling in the four directions of east, south, west, and north, and eight leaves from each seedling. The samples were stored in a refrigerator at -80 °C to further determine antioxidant enzyme activity, MDA content, osmoregulatory substance content, and photosynthetic pigment content.

### Determination of antioxidant enzyme activity and MDA content

To determine the activities of POD and SOD in each sample, the methods outlined by Li [27] and Beauchamp and Fridovich [28] were followed. Samples (0.5 g) were pulverized, and 10 mL of phosphate-buffered saline (PBS) at a concentration of 0.05 mol/L was added. Subsequently, the samples were centrifuged at 12, 000 rpm for 15 min, and the resulting supernatant was collected. Following this, a reaction solution consisting of 28 μL of guaiacol and 19 μL of 30% H_2_O_2_ in PBS was mixed with 1 mL of the extraction solution, and POD activity was determined by measuring the optical density (OD) at 470 nm. To assess the SOD activity, a solution containing 1.5 mL of PBS with a concentration of 0.05 mol/L, 0.3 mL of Met (130 mmol/L) with a concentration of 0.13 mol/L, 0.3 mL of nitro blue tetrazolium with a concentration of 0.75 μmol/L, 0.3 mL of EDTA-Na_2_ (100 μmol/L) with a concentration of 0.1 μmol/L, 0.5 mL of distilled water, and 0.3 mL of riboflavin with a concentration of 0.02 μmol/L was added to 0.1 mL of the supernatant. The resulting solution was then subjected to OD measurement at 560 nm.

CAT activity was determined according to the protocols outlined by Li [27] and Díaz-Vivancos [29]. In brief, a mixture of 2.9 mL of reaction solution (comprising 0.155 mL of 30% H_2_O_2_ in PBS) and 0.1 mL of extract was prepared. Subsequently, the optical density (OD) of the resulting solution was measured at 240 nm wavelength.

The MDA content was measured using the thiobarbituric acid (TBA) method with some modifications [27]. Samples (0.5 g each) were homogenized in 10 mL 10% trichloroacetic acid (TCA). The homogenate was centrifuged at 4000 r/min for 10 min. Next, 2 mL of the supernatant and 2 mL of 10% TCA containing 0.6% (w/v) TBA were added. The homogenate was boiled for 15 min, cooled in an ice bath, and centrifuged at 3000r/min for 15 min. The absorbance of the supernatant was measured at 450 nm, 532 nm, and 600 nm. The MDA content was calculated using the following formulas:


$${\text{C}}_{\text{MDA}}\text{=6}.45\times \left({\text{A}}_{\text{532}}{\text{-A}}_{\text{600}}\right)\text{-0}{\text{.56×A}}_{\text{450}}$$ (a)

$$\text{MDA content }\left(\text{μmol/g Fw}\right){\text{=C}}_{\text{MDA}}\text{×V/ }\left({\text{V}}_{1}\text{×W}\right)$$ (b)

Where C_MDA_ is the concentration of MDA in the reaction mixture (μmol/ml); V is the total volume of extract (ml); V_1_ is the volume of extract taken for the determination (ml); W is the fresh weight of the sample (g); A_450_, A_532_ and A_600_ is the absorbance of the supernatant was measured at 450 nm, 532 nm, and 600 nm.

### Determination of osmoregulatory substance content

The SS and St contents were estimated using the method described by Li [27], with some modifications. Took the sample 0.5 g, grind it with distilled water and made it into 10 ml, put it into boiling water for 30 min, cooled it down to room temperature, then centrifuged it at 12000 r/min for 20 min, took up the supernatant into tube A, added distilled water to the centrifuge tube with residue and made it into 10 ml, centrifuged it at 12000 r/min for 20 min, the supernatant was transferred to tube A again, and was fixed to 25 ml, shaken well, and used for the determination of SS. The assay solution contained the extraction solution (0.5 mL), distilled water (1.5 mL), anthrone ethyl acetate (0.5 mL), and 98% H_2_SO_4_ (5 mL). A HitachiU-5100 UV spectrophotometer (Hitachi, Tokyo, Japan) was used to measure the OD to determine the amount of SS at 630 nm. The SS residue was added to the centrifuge tube, and distilled water was added until a total volume of 10 mL was attained. To extract the resulting solution, it was boiled in water for 30 minutes. Subsequently, an additional 2 mL of perchloric acid with a concentration of 9.2 mol/L was added. Extraction was repeated for 20 min, utilizing the same method that was employed for SS, to ascertain the St content.

The methods of Li [27] and Bradford [30] were used to determine the SP content, with some modifications. The sample (0.5 g) was added to distilled water, ground until homogenized, transferred to a 10 ml centrifuge tube, and the volume was made up to 10 ml with distilled water. The supernatant was the protein extract, which was centrifuged it at 12000 r/min for 20 min. Pipette 1 ml of protein extract was transferred into a test tube, add 5 ml of Coomassie Brilliant Blue G-250 reagent and mix well. After allowing to stand for 2 min, the SP content was measured by determining OD at 595 nm.

The proline content was estimated using the method described by Li [27], with some modifications. Each sample (0.5 g each) was added to 5 mL of 3% sulfosalicylic acid solution, extracted in boiling water for 10 min, and the supernatant was cooled to room temperature. Then, 2 mL of supernatant, 2 mL of glacial acetic acid, and 2 mL of acidic hydrated ninhydrin were added to develop a colored solution in the test tube. The solution was boiled in a water bath for 30 min, and then cooled. To each sample, 4 mL of toluene was added, and the solution was oscillated for 30 s and left to stand so that all pigment was transferred to the toluene solution. The upper layer of the proline toluene solution was aspirated from each tube and the OD was measured at 520 nm using toluene solution as the blank control.

### Determination of photosynthetic pigment content

The Chl and Car were extracted using the acetone extraction method described by Lichtenthaler [31] (1987), with some modifications; acetone was used as a control. Using a HitachiU-5100 UV spectrophotometer (Hitachi), the OD was measured at 440, 645, and 663 nm.

The Chl and Car contents were calculated using the following formulas:


$${\text{C}}_{\text{chl a}}\text{=9}{\text{.78 OD}}_{\text{663}}\text{-0}{\text{.99 OD}}_{\text{645}}$$ (a)

$${\text{C}}_{\text{chl b}}\text{=21}{\text{.43 OD}}_{\text{645}}\text{-4}{\text{.65 OD}}_{\text{663}}$$ (b)

$${\text{C}}_{\text{chl}}\text{=5}{\text{.13 OD}}_{\text{663}}\text{-20}{\text{.44 OD}}_{\text{645}}$$ (c)

$${\text{C}}_{\text{car}}{\text{=470 OD}}_{\text{440}}\text{-0}{\text{.27 C}}_{\text{chl}}$$ (d)

$$\text{A=C×V/}\left(\text{m×1000}\right)$$ (e)

Where C is the mass concentration of the pigment (mg/L), A is the mass fraction of the pigment (mg/g), V is the volume of the extract (mL), and m is the fresh weight of the leaf (g).

### Determination of photosynthetic parameters

Pn, Tr, Gs, and Ci were measured using a Li-6400XT portable photosynthesizer (LI-COR Inc., Lincoln, NE, USA) during June, July, and August 2022, when the weather was sunny. Mature leaves were collected from the middle of the branches of each seedling and three replicates were measured for each line. The first leaves sampled were numbered and labeled for subsequent analyses.

### Data analysis

Data on growth traits, antioxidant enzyme activity, MDA content, osmoregulatory substance content, photosynthetic pigment content, and photosynthetic parameters were analyzed using SPSS 26.0 (IBM Corp., Armonk, NY, USA). Independent-samples T-tests were used to compare the means and significance of the treatment and control groups. Bar graphs were created using Excel 2019 software (Microsoft, Redmond, Washington, USA), and the data were displayed as the mean±standard deviation of the mean (SD). Additionally, we constructed a correlation heat map using Origin Pro 2021 software (EA, Northampton, Massachusetts, USA).

## Results

### Growth traits

**Fig 1:**
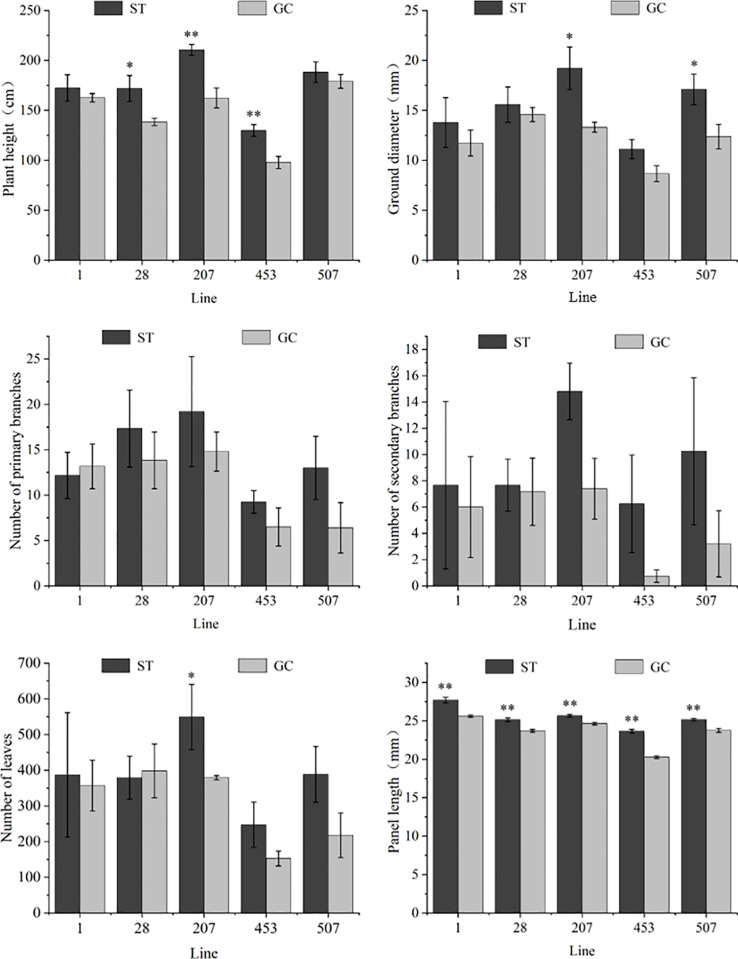
Changes in growth index among different lines of spaceflight treatment *of P. sibirica* seedlings. Data are presented as mean ± SD. ** indicates that the difference is extremely significant at the 0.01 level, and * indicates that the difference is significant at the 0.05 level.

As shown in Fig 1, growth traits were altered in all lines after spaceflight treatment compared with the ground control, and the degree of response of different lines to spaceflight treatment varied. Compared with GC seedlings, the plant height of ST28, ST207, and ST453 seedlings was significantly increased by 24.14%, 29.68%, and 32.17%, respectively, whereas the difference in plant height between ST1 and ST507 seedlings did not reach a significant level. The ground diameter of ST207 and ST507 seedlings was significantly higher than that of GC207 and GC507 seedlings, while the ground diameter of ST1, ST28, and ST453 seedlings did not change significantly compared to that of GC seedlings. The number of primary branches of the ST1 seedlings was lower than that of the GC1 seedlings, but the number of primary branches of the ST28, ST207, ST453, and ST507 seedlings and the number of secondary branches of each line were higher than those of the GC seedlings, and the differences were not significant. The number of leaves in ST207 seedlings was significantly higher than that in GC207 seedlings, with an increase of 44.78%, however there was no significant change in any of the other lines. The panel length of all lines in the spaceflight treatment was significantly higher than that of the GC seedlings, and the panel length of ST453 seedlings showed the greatest increase, which was 16.60% higher than that of the GC453 seedlings.

### Leaf morphology

**Fig 2:**
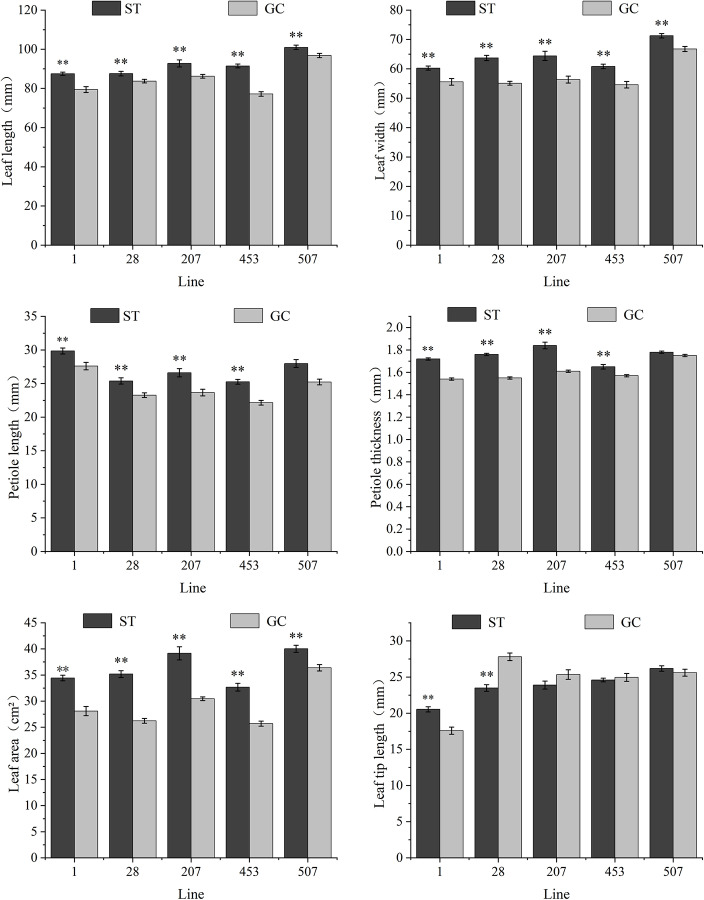
Changes in leaf morphology in different lines of spaceflight treatment of *P. sibirica* seedlings. Data are presented as mean ± SD. ** indicates that the difference is extremely significant at the 0.01 level, and * indicates that the difference is significant at the 0.05 level.

As shown in Fig 2, the coefficient of variation in the leaf morphology indices within the population of each line was small. Compared to the GC seedlings, the leaf length, leaf width, and leaf area of each line after spaceflight treatment were significantly increased by 4.62%-18.47, 6.72%-15.64%, and 9.97%-34.18. Petiole length and petiole thickness were significantly higher in ST1, ST28, ST207, and ST453 seedlings, but there was no significant change in ST507 seedlings compared with GC seedlings. The leaf tip length of each line treated with spaceflight changed in different directions, and the leaf tip length of ST1 seedlings was significantly higher than that of GC1 seedlings by 16.83%, whereas that of ST28 seedlings was significantly lower than that of GC28 seedlings by 15.54%. The leaf tip lengths of the ST207, ST453, and ST507 seedlings did not show any significant changes.

### Antioxidant enzyme activities and MDA content

**Fig 3:**
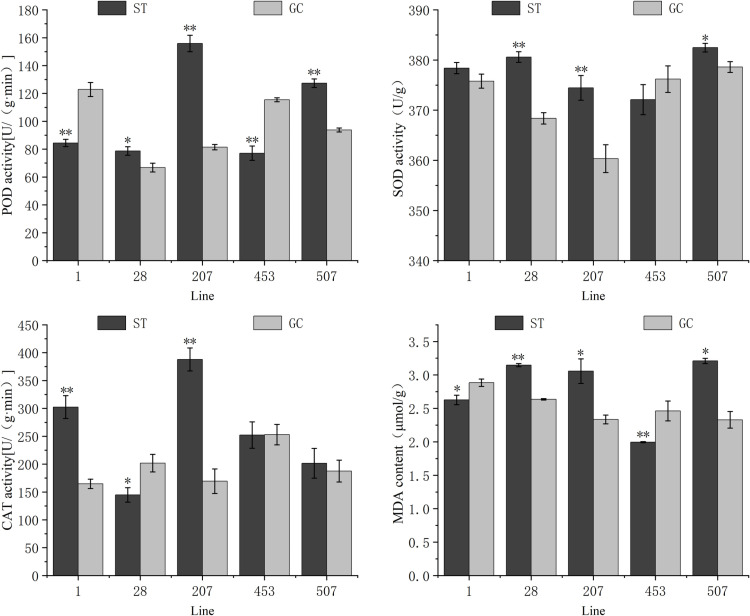
Changes in antioxidant enzyme activity and MDA levels in different lines of spaceflight treatment *of P. sibirica* seedlings. Values are presented as the mean ± SD. ** indicates that the difference is extremely significant at the 0.01 level, and * indicates that the difference is significant at the 0.05 level.

As shown in Fig 3, compared with the ground control, significant changes in antioxidant enzyme activities and MDA content were observed in *P. sibirica* seedlings after spaceflight treatment. The different lines and indicators responded to spaceflight treatment to varying extents. The POD activities of ST1 and ST453 seedlings were 31.28% and 33.21% lower than those of GC1 and GC453 seedlings, respectively. The POD activities of ST28, ST207, and ST507 seedlings were significantly or very significantly higher than those of GC seedlings, with those of ST207 seedlings being 1.91 times higher than those of GC207 seedlings. The SOD activity of ST453 seedlings was significantly lower than that of GC453 seedlings, the SOD activity of ST28, ST207, and ST507 seedlings was significantly higher than that of GC seedlings, and the SOD activity of ST1 seedlings did not reach a significant level in comparison with that of GC1 seedlings. After spaceflight treatment, the CAT activity of ST1 and ST207 seedlings was significantly higher than that of GC seedlings, reaching 1.84 and 2.28 times of GC1 and GC207 seedlings, respectively, whereas that of ST28 seedlings was significantly lower than that of GC28 seedlings by 28.25%. The CAT activities of the ST453 and ST507 seedlings were not significantly different from those of the GC seedlings. The MDA contents of ST28, ST207, and ST507 seedlings were significantly or highly significantly higher than those of GC seedlings, and the MDA contents of ST1 and ST453 seedlings were significantly lower than those of GC1 and GC453 seedlings.

### Osmoregulatory substances content

**Fig 4:**
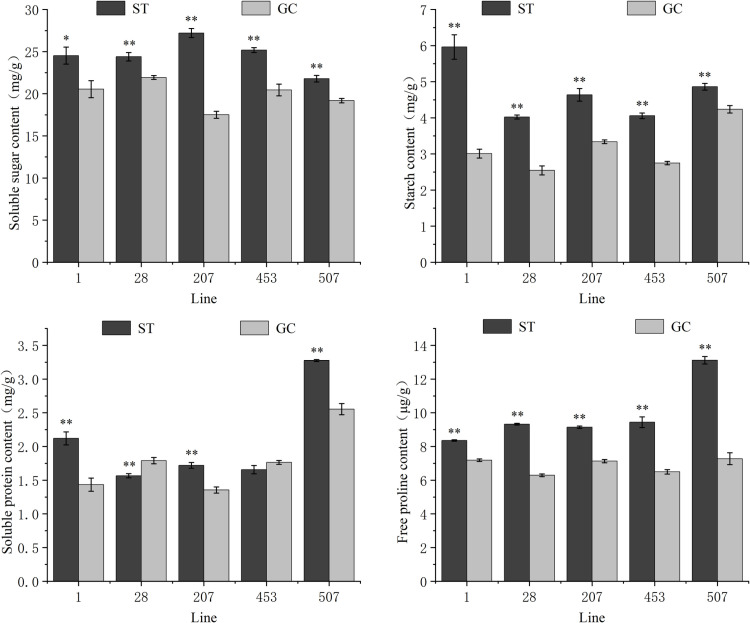
Changes in osmoregulatory substance content in different lines of spaceflight treatment in *P. sibirica* seedlings. Values are presented as the mean ± SD. ** indicates that the difference is extremely significant at the 0.01 level, and * indicates that the difference is significant at the 0.05 level.

As shown in Fig 4, compared with the GC seedlings, the SS content of each line after spaceflight treatment increased by 19.36%, 11.23%, 55.40%, 23.14%, and 13.53%, respectively. All of these values reached a highly significant level. The St content of each line after spaceflight treatment was 14.76%-98.18% higher than that of GC seedlings, and the St content of ST1 seedlings was 5.96 mg/g, which was 1.98 times higher than that of GC1 seedlings. The SP content of each line changed significantly after spaceflight treatment. The SP content of ST1, ST207, and ST507 seedlings were significantly higher than that of GC seedlings, with increases of 47.89%, 26.92%, and 28.23%, respectively. The SP content of ST28 and ST453 seedlings was lower than that of GC28 and GC453 seedlings, with the difference in ST28 seedlings being significantly lower than in GC28 seedlings by 12.50%, but the difference in ST453 seedlings did not reach a significant level. The Pro content of each line after spaceflight treatment was significantly higher than that of GC seedlings, with an increase of 16.32%-80.38%, among which the Pro content of ST507 seedlings (13.12 μg/g) was the highest, and the increase was 1.80 times that of GC507 seedlings.

### Photosynthetic pigment content

**Fig 5:**
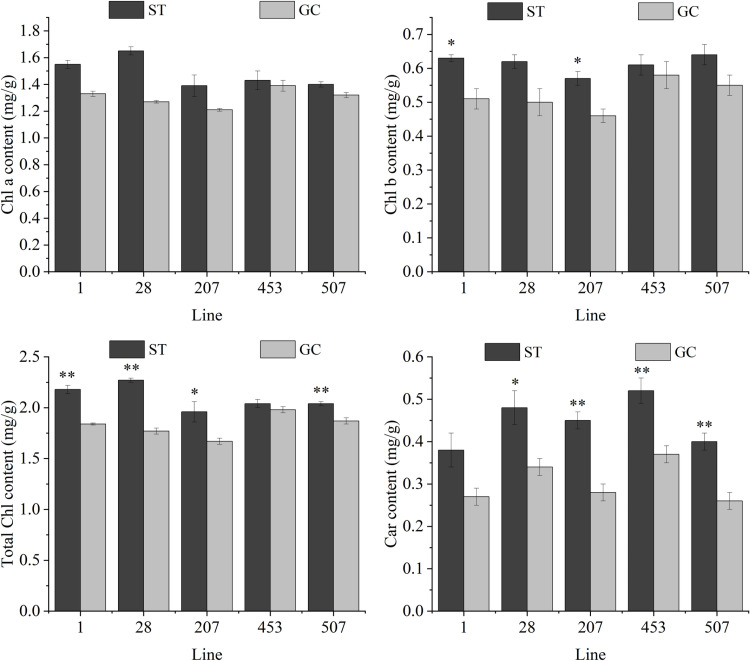
Changes in photosynthetic pigment content in different lines of spaceflight treatment in *P. sibirica* seedlings. Values are presented as the mean ± SD. ** indicates that the difference is extremely significant at the 0.01 level, and * indicates that the difference is significant at the 0.05 level.

Changes in photosynthetic pigment content of leaves of *P. sibirica* seedlings in different degrees after spaceflight treatment (Fig 5). The Chl a content of ST1 and ST28 seedlings was 16.72% and 30.45% higher than that of GC seedlings, respectively, and the Chl a content of ST207, ST453, and ST507 seedlings slightly increased without reaching a significant level. Compared with GC seedlings, the Chl b content increased in ST1, ST28, ST 207, ST453, and ST507 seedlings and reached a significant level in ST1 and ST28 seedlings, but the difference was not significant in ST207, ST453, and ST507 seedlings. The Chl content of ST1, ST28, ST207, and ST507 seedlings were 18.47%, 28.63%, 17.44%, and 8.66%, respectively, which were significantly higher than those of GC seedlings, whereas there was no significant change in ST453 seedlings. Compared with GC seedlings, the Car content of ST28, ST207, ST453, and ST507 seedlings was 41.18%, 61.26%, 41.23%, and 54.55%, respectively, higher than that of GC seedlings, while there was no significant change in ST1 seedlings.

### Photosynthetic parameters

**Fig 6:**
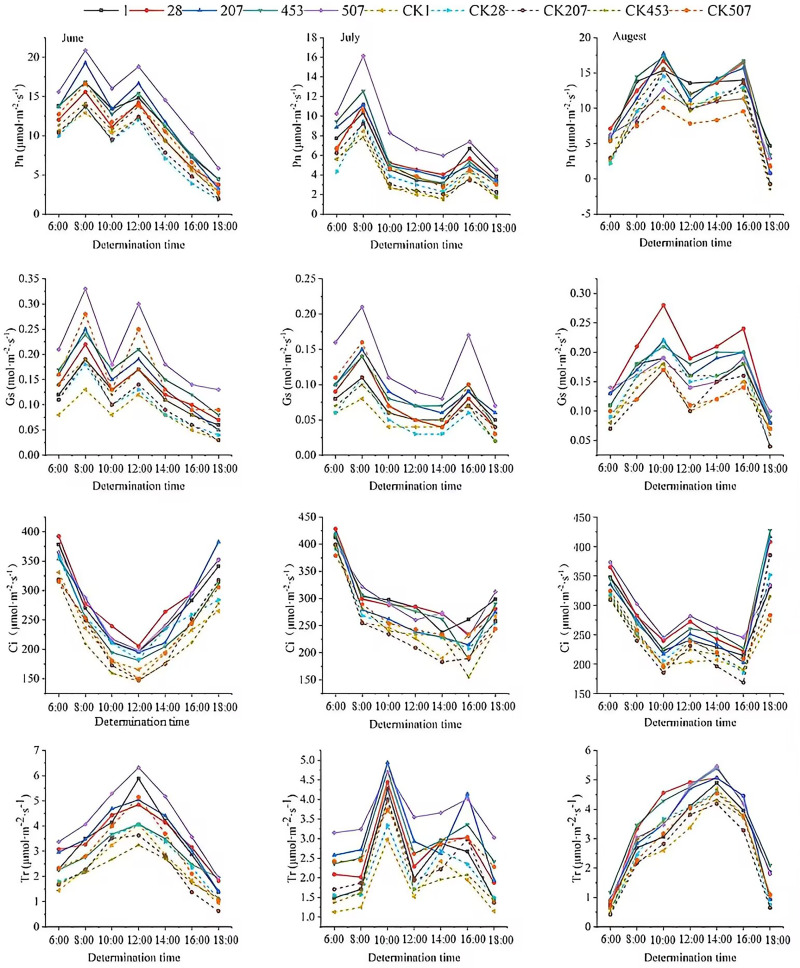
Changes in photosynthetic indices in different lines of spaceflight treatment seedlings of *Prunus sibirica.*

As shown in Fig 6, compared with GC seedlings, the Pn, Gs, Tr and Ci of each line were significantly increased by the spaceflight treatment. The daily changes in the Pn rate of *P. sibirica* seedlings in June, July, and August were bimodal, with the “photosynthesis lunch break” phenomenon observed in all five lines. The peaks appeared at slightly different times in different months, with the first peak occurring at 8:00 a.m. in June and July, and at 10:00 a.m. in August. Compared to the GC seedlings, the peaks of the seedlings after spaceflight treatment increased by 13.39–40.95% in June, 17.98–59.66% in July, and 10.47–33.33% in August. The second peak appeared at 16:00 in July and August, whereas it appeared at 12:00 in June, and the second peak of SP207 seedlings increased by 84.32% in July compared to that of GC seedlings. The Pn rate of the ST507 seedlings in June and July was significantly higher than that of the other lines at all time points; however, the difference in the Pn rate of each line was not significant in August.

The trend of Gs changed in the seedlings of the five lines in June, July, and August was consistent with the changes in Pn at the time of the peaks. The Gs in June was slightly higher than that in July and August. The daily changes in Tr in June and August peaked at 12:00 and 14:00, respectively. The daily changes in Tr in July doubled, with the first peak occurring at 10:00 a.m., but the time of the second peak appeared differently in different lines, with the second peak appearing at 14:00 in the case of ST1, GC1, and GC28 seedlings, and at 16:00 in the case of the remaining lines. Ci in seedlings of all lines after spaceflight treatment was higher than that of GC seedlings and showed a “V” curve with a decreasing and then increasing trend in June and July, while a “W” curve with a decreasing and then increasing trend with a decreasing and then increasing trend was observed in August.

### Correlation analysis of indexes

**Fig 7:**
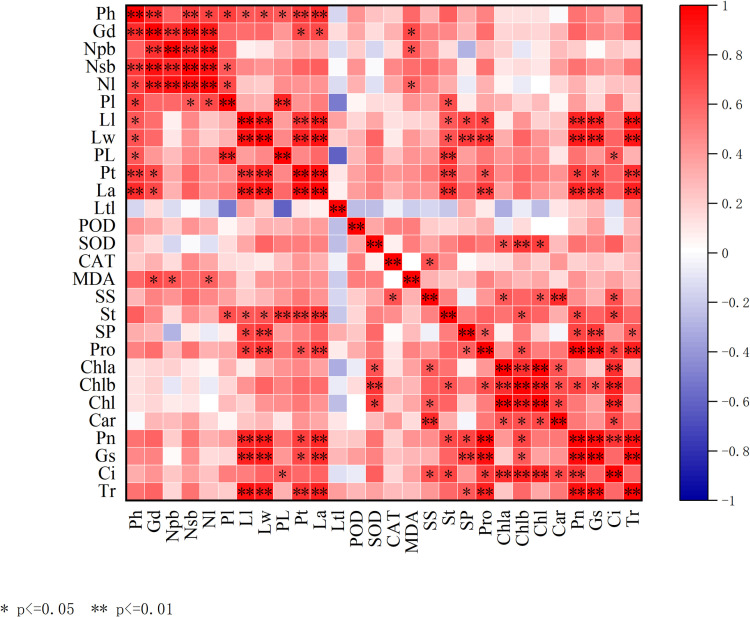
Correlation between growth traits and physiological indices of *Prunus sibirica* seedlings after spaceflight treatment. Red indicates a positive correlation and blue indicates a negative correlation. The color intensity was proportional to the correlation coefficient. Ph. Plant height, Gd. Ground diameter, Np. Number of primary branches, Ns. numbers of secondary branches (Nl. Number of leaves, Pl. Panel length, Ll. Leaf length, Lw. Leaf width, PL. Petiole length, Pt. Petiole thickness La. Leaf area, Ltl. Leaf tip length.

Correlation analysis of the growth characteristics, leaf morphology, antioxidant enzyme activity, osmoregulatory substance content, pigment content, and photosynthetic indices of each line after spaceflight treatment (Fig 7). Plant height, ground diameter, and leaf number were significantly or highly significantly positively correlated, whereas leaf length, leaf width, petiole thickness, and leaf area were significantly or highly significantly positively correlated. Plant height was significantly or highly significantly correlated with leaf length, leaf width, petiole length, petiole thickness, leaf area, ground diameter, number of primary branches, and MDA content, with a correlation coefficient greater than 0.600. The leaf length, width, and area were significantly or highly positively correlated with St, Pro, Pn, Gs, and Tr. Ci was significantly negatively correlated with the number of secondary branches, petiole thickness, and leaf area. St, SP, and Pro contents were significantly or highly significantly positively correlated with Pn, and SS content was significantly or highly significantly positively correlated with Chl a, Chl, and Car, and significantly negatively correlated with Ci. Car, Chl, Chl a, and Chl b content showed highly significant positive correlations with each other. Chl b levels were significantly positively correlated with Pn and Gs. Pn, Gs, and Tr showed a highly significant positive correlation, and Ci showed a highly significant positive correlation with Pn.

## Discussion

### Changes in plant growth traits after spaceflight treatment

Unlike terrestrial environments, space conditions could affect plant growth, physiology, biochemistry, and genetics, leading to abundant changes in the plant [8, 32–35]. Compared to ground control, the plant height and ground diameter of *Acer mono* [14] and *Avena sativa* [23] raised from spaceflight seeds were increased, whereas those *of Robinia pseudoacacia* [23] and *Betula platyphylla* [24] were decreased. In the present study, plant height and ground diameter of *P. sibirica* seedlings raised from spaceflight seeds were higher than those of GC seedlings, but different lines showed different degrees of alteration. The plant height and ground diameter of ST 207 seedlings were significantly or extremely significantly higher than those of GC 207 seedlings, whereas the differences in the other lines did not reach significance. These results indicated that the seedlings raised from spaceflight seeds maybe induce high-frequency genetic variation, and the response patterns of different plant species to spaceflight treatments vary significantly. Branch and leaf numbers are important agronomic traits and photosynthetic organs in plants, and are key factors in plant size, yield, and biomass [36–38]. There was no significantly change in the number of leaves and branches of *P. sibirica* seedlings raised from spaceflight seeds. However, the number of leaves and branches increased significantly in *Cassia obtusifolia* that have been treated through spaceflight [15], which was presumed to be due to the uncertainty of the spaceflight treatment as well as the existence of genetic differences among the lines [10].

### Changes in leaf morphology after spaceflight treatment

Leaf morphology, including leaf size, shape, color, petiole size, and other important indicators for plant taxonomic identification, is often used to directly reflect plant adaptation to different environments [39]. Environmental changes can cause changes in the leaf morphology of plants [40,41]. In the present study, the leaf size was greater than that of GC seedlings, and longer petioles were formed in *P. sibirica* seedlings raised from spaceflight seeds, which could help regulate the spatial distribution of leaves and improve the ability of the plant to acquire and utilize light energy [42]. The size of the leaf area determined the size of the plant’s photosynthetic area, and the leaf area of *P. sibirica* seedlings raised from spaceflight seeds increased significantly compared to GC seedlings, which is consistent with the results of Queshe, Rougui, and Qidan after spaceflight treatment [43]. It had been hypothesized that spaceflight treatment induces chromosomal aberrations and mutations in plant seeds, causing chromosomal and genetic mutations in germ cells and leading to changes in leaf traits in plant monocultures.

### Changes in antioxidant enzyme activities after spaceflight treatment

Specialized space environments may be an obstacle for plants on Earth compared with terrestrial environments [11]. Plants subjected to adverse stresses caused by cell membrane lipid peroxidation generate large amounts of free radicals and ROS [44–46]. To prevent detrimental effects, the autochthonous protective enzyme system of the plant is activated. POD, SOD, CAT, and other protective enzymes played important roles in maintaining normal cellular metabolism [47–49]. Compared with GC seedlings, the activities of antioxidant enzymes, POD, SOD, and CAT, in various lines of *P. sibirica* seedlings raised from spaceflight seeds showed different degrees of change, it is hypothesized that this may be due to the influence of the complex spatial environment on the growth of *P. sibirica*. The seedlings of various lines defended against damage caused by reactive in plant cells by altering the activity of antioxidant enzymes [14]. Membrane lipid peroxidation ultimately produces MDA, and the MDA content is generally regarded as an important indicator of the degree of cellular damage. High MDA content indicated severe cellular damage and membrane lipid peroxidation [11, 50]. The MDA content significantly or extremely significantly increased in ST28, ST207, and ST507 seedlings compared with GC seedlings. This may predict that the complex environment of space has some effect on *P. sibirica*, and these three lines are sensitive to the effects of the spatial environment, resulting in the accumulation of MDA content in the leaves and an increase in the degree of cellular membrane plasma peroxidation [11].

### Changes in osmoregulatory substances content after spaceflight treatment

Osmoregulation is a self-developed defence mechanism in plants under stressful conditions [51]. SP, Pro, SS, and St contents were important osmoregulatory substances in plants. An increase or decrease in their content helped regulate the osmotic pressure inside and outside the cell to maintain normal cellular metabolism, and their elevated content is conducive to plant growth and improved the ability of plants to adapt to stress. Song showed that the SP content in *Populus ussuriensis* increased after spaceflight treatment, and cellular osmoregulation ability was enhanced, demonstrating strong resistance to stress [11]. The SP, SS, St, and Pro contents in the leaves of *P. sibirica* seedlings raised from spaceflight seeds were significantly higher than those in the GC seedlings. Owing to the extremely complex environmental conditions in space, many factors exist, and it is difficult to determine the dominant factors that often lead to uncertainty in the discrepancy results of spaceflight. It tentatively hypothesizes that this is due to the fact that spaceflight treatment promotes the accumulation of osmoregulatory substances in plants, which helped to increase the concentration of cytosol, maintain cell expansion pressure, and enhance the water retention capacity of cells, thus improving plant stress tolerance [52,53].

### Changes in the photosynthetic pigment content after spaceflight treatment

Chloroplasts are extremely sensitive to environmental stresses during photosynthesis [54,55]. The structural integrity of chloroplasts is important for changes in the pigment content, plant growth, and development. Chloroplasts are sites of light and dark reactions in plants and contain large amounts of photosynthetic pigments such as Chl, Chl a, Chl b, and Car. Chl plays an important role in the absorption, transportation, and conversion of light energy in plants. Car served as a protective agent and plays an important role in the light-harvesting mechanism of photosynthetic plants by protecting them from ROS [56]. The Chl and Car content in *Xanthoceras sorbifolium*, *Robinia pseudoacacia*, and *Acer mono* raised from spaceflight seeds were lower than GC seedlings, but the leaf pigment content of *P. sibirica* seedlings and Rougui [43] were higher than GC seedlings. Higher Chl content facilitates photosynthesis, resulting in stronger plants and subsequently, higher yields. It is assumed that the sensitivity of the enzymes involved in Chl synthesis to spaceflight transport varies between species, and the Chl a and Chl b content were varied owing to differences in metabolic pathways.

### Changes in the photosynthetic parameters after spaceflight treatment

Photosynthesis is necessary for plant growth and development and drives its life-processing cycle [57]. Photosynthesis is also able to influence the fruiting of economic forest species, which governs the level of yield and is the basis for yield formation [58]. The Pn rate directly indicated the photosynthetic capacity of the leaves [59], The higher the Pn rate, the more fully the plant is able to utilize light energy for photosynthesis and produce more organic matter and energy, thus increasing yield. the Tr reflected the transpiration intensity of the plants, the Gs indicated the degree of stomatal opening and the Ci indicated the capacity of plant chloroplasts to assimilate CO_2_, which is an important indicator of plant photosynthesis and is closely related to the Pn rate [60]. The Pn rate, Gs, Tr and Ci of *P. sibirica* seedlings after spaceflight treatment were significantly higher than those of GC seedlings. It was hypothesized that spaceflight treatment may have induced changes in stomatal distribution and shape characteristics of the leaves, resulting in increased stomatal opening and Gs, which led to elevated Tr and Ci, which in turn led to an elevated Pn rate [14]. Because there are many uncertainties associated with spaceflight treatment, the accuracy of this conjecture must be verified in many ways. In this study, we found that the Pn rate of ST507 was significantly higher than that of other lines, and it might be that its yield was also better than that of other lines, and it could be used as a potential high-yielding strain for selection and breeding.

In summary, compared to GC, the growth traits, antioxidant enzyme activities, osmoregulatory substances, photosynthetic pigment content and photosynthetic parameters were changed in *P. sibirica*. seedlings raised from spaceflight seeds. However, owing to the extremely complex and special space environment and many physical factors, the factors that play a dominant role in causing *P. sibirica*. seedlings raised from spaceflight seeds to differ from ground control need to be further investigated in the future. Previous studies have shown that the growth of seedlings grown from spaceflight seeds may be superior to that of terrestrial controls, but this advantage may gradually increase, weaken, or even disappear with growth [11, 13]. In the present study, all the indices of *P. sibirica* seedlings grown from seeds treated by spaceflight changed compared with ground control, and whether this phenotypic advantage is stable requires further observation and investigation at the molecular level in future studies. Meanwhile, research on screening and asexual propagation technology of differential strains of potentially spaceflight-mutated *P. sibirica* should be conducted in the future to help fix and effectively utilize beneficial alterations through asexual propagation technology.

## Conclusion

In conclusion, compared with the control, the height, ground diameter, internode length and leaf morphology of *P. sibirica* seedlings raised from spaceflight seeds were changed more, but the variations on branching and leaf number were smaller. Meanwhile, antioxidant enzyme activities, osmoregulatory substance contents and photosynthetic pigment contents in all lines of *P. sibirica* changed to different degrees after spaceflight treatment. The photosynthetic rate, stomatal conductance, intercellular CO_2_ concentration, and transpiration rate were higher than those of the ground control in all lines treated with spaceflight. Among them, the photosynthetic capacity of 507 was particularly outstanding, and it can be used as a potential high product line.

## Supporting information

S1 Table Changes in growth index among different lines of spaceflight treatment of *Prunus sibirica* seedlings. (DOCX)

S2 Table Changes in leaf morphology in different lines of spaceflight treatment of *Prunus sibirica* seedlings. (DOCX)

S3 Table Changes in antioxidant enzyme activity and MDA levels in different lines of spaceflight treatment of *Prunus sibirica* seedlings. (DOCX)

S4 Table Changes in osmoregulatory substance content in different lines of spaceflight treatment in *Prunus sibirica* seedlings. (DOCX)

S5 Table Changes in photosynthetic pigment content in different lines of spaceflight treatment in *Prunus sibiric*a seedlings. (DOCX)
