# Supplementary material for: Alterations in physiological and biochemical characteristics of Prunus sibirica seedlings raised from spaceflight seeds
Source: PLoS One. 2025 Apr 24;20(4):e0321147. doi: 10.1371/journal.pone.0321147 (PMC12021159; doi:10.1371/journal.pone.0321147)
Supplement: S1 Table — (DOCX) [file pone.0321147.s001.docx]

**Supporting Information captions**

**S1 Table Changes in growth index among different lines of spaceflight treatment of *Prunus sibirica* seedlings.**

| Line | Plant height | | Ground diameter | | Number of primary branches | | Number of secondary branches | | Number of leaves | | Panel length | |
| --- | --- | --- | --- | --- | --- | --- | --- | --- | --- | --- | --- | --- |
|  | ST | GC | ST | GC | ST | GC | ST | GC | ST | GC | ST | GC |
| 1 | 172.42±13.23 Aa | 162.62±4.23 Aa | 13.79±2.49 Aa | 11.73±1.30 Aa | 12.17±2.54 Aa | 13.17±2.24 Aa | 7.67±6.37 Aa | 6.00±3.84 Aa | 387±174.44 Aa | 357.17±71.18 Aa | 27.70±0.37 Aa | 25.62±0.15 Bb |
| 28 | 171.82±12.98 Aa | 138.40±3.68Ab | 15.58±1.76 Aa | 14.58±0.71 Aa | 17.33±4.24 Aa | 13.83±3.13 Aa | 7.67±1.98 Aa | 7.17±2.56 Aa | 379.17±60.27 Aa | 398.17±75.29 Aa | 25.14±0.24 Aa | 23.71±0.18 Bb |
| 207 | 210.62±5.38 Aa | 162.62±9.99 Bb | 19.21±2.14 Aa | 13.31±0.50 Ab | 19.20±6.06 Aa | 14.80±2.15 Aa | 14.80±2.15 Aa | 7.40±2.32 Aa | 549.00±91.51 Aa | 379.20±5.89 Ab | 25.67±0.17 Aa | 24.64±0.18 Bb |
| 453 | 129.83±5.88 Aa | 97.83±6.08 Bb | 11.12±0.95 Aa | 8.67±0.80 Aa | 9.25±1.25 Aa | 6.50±2.10 Aa | 6.25±3.71 Aa | 0.75±0.48 Aa | 247.50±63.39 Aa | 152.75±20.79 Aa | 23.65±0.25 Aa | 20.28±0.18 Bb |
| 507 | 188.36±10.25 Aa | 179.08±6.92 Aa | 17.10±1.53 Aa | 12.37±1.23Ab | 13.00±3.48 Aa | 6.40±2.77 Aa | 10.25±5.60 Aa | 3.20±2.52 Aa | 388.75±78.14 Aa | 217.80±62.74 Aa | 25.15±0.17 Aa | 23.76±0.26 Bb |

Note: Data are presented as mean ± SD. Large letter indicates that the difference is extremely significant at the 0.01 level, and small letter indicates that the difference is significant at the 0.05 level.
