# Supplementary material for: Alterations in physiological and biochemical characteristics of Prunus sibirica seedlings raised from spaceflight seeds
Source: PLoS One. 2025 Apr 24;20(4):e0321147. doi: 10.1371/journal.pone.0321147 (PMC12021159; doi:10.1371/journal.pone.0321147)
Supplement: S2 Table — (DOCX) [file pone.0321147.s002.docx]

**Supporting Information captions**

**S2 Table Changes in leaf morphology in different lines of spaceflight treatment of *Prunus sibirica* seedlings.**

| Line | Leaf length | | Leaf width | | Petiole length | | petiole thickness | | Leaf tip length | | Leaf area | |
| --- | --- | --- | --- | --- | --- | --- | --- | --- | --- | --- | --- | --- |
|  | ST | GC | ST | GC | ST | GC | ST | GC | ST | GC | ST | GC |
| 1 | 87.49±0.83 Aa | 79.41±1.51 Bb | 60.25±0.69 Aa | 55.57±1.12 Bb | 29.85±0.44 Aa | 27.60+0.56 Bb | 1.72±0.01 Aa | 1.54±0.01 Bb | 20.54±0.36 Aa | 17.58±0.50 Bb | 34.43±0.36 Aa | 28.10±0.50 Bb |
| 28 | 87.58±1.16 Aa | 83.71±0.92 Bb | 63.71±0.88 Aa | 55.09±0.65 Bb | 25.39±0.46 Aa | 23.27±0.35 Bb | 1.76±0.01 Aa | 1.55±0.01 Bb | 23.48±0.46 Bb | 27.80±0.52 Aa | 35.19±0.46 Aa | 26.23±0.52 Bb |
| 207 | 92.77±1.79 Aa | 86.19±0.95 Bb | 64.41±1.56 Aa | 56.33±1.17 Bb | 26.61±0.62 Aa | 23.65±0.49 Bb | 1.84±0.03 Aa | 1.61±0.01 Bb | 23.89±0.55 | 25.34±0.66 | 39.16±0.55 Aa | 30.46±0.66 Bb |
| 453 | 91.45±0.97 Aa | 77.19±1.17 Bb | 60.82±0.77 Aa | 54.59±1.10 Bb | 25.26±0.36 Aa | 22.15+0.35 Bb | 1.65±0.02 Aa | 1.57±0.01 Bb | 24.59±0.26 | 24.95±0.54 | 32.67±0.26 Aa | 25.70±0.54 Bb |
| 507 | 100.96±1.10 Aa | 96.82±1.08 Bb | 71.26±0.74 Aa | 66.77±0.86 Bb | 27.98±0.58 | 25.23±0.43 | 1.78±0.01 | 1.75±0.01 | 26.18±0.37 | 25.60±0.48 | 40.02±0.37 Aa | 36.39±0.48 Bb |

Note: Data are presented as mean ± SD. Large letter indicates that the difference is extremely significant at the 0.01 level, and small letter indicates that the difference is significant at the 0.05 level.

**Table S3 Changes in antioxidant enzyme activity and MDA levels in different lines of spaceflight treatment *of Prunus sibirica* seedlings*.***

| Line | POD activity | | SOD activity | | CAT activity | | MDA content | |
| --- | --- | --- | --- | --- | --- | --- | --- | --- |
|  | ST | GC | ST | GC | ST | GC | ST | GC |
| 1 | 84.44±2.58 Bb | 122.89±5.04 Aa | 378.39±1.12 Aa | 375.79±1.39 Aa | 302.46±20.42 Aa | 164.58±8.44 Bb | 2.63±0.07 Bb | 2.88±0.05 Aa |
| 28 | 78.67±3.04 Aa | 66.78±3.12 Ab | 380.60±1.07 Aa | 368.36±1.13 Bb | 144.79±13.02 Ab | 201.79±15.77 Aa | 3.15±0.02 Aa | 2.63±0.01 Bb |
| 207 | 155.89±5.94 Aa | 81.44±1.95 Bb | 374.45±2.46 Aa | 360.33±2.77 Bb | 387.83±20.64 Aa | 169.46±22.00 Bb | 3.06±0.18 Aa | 2.34±0.07 Ab |
| 453 | 77.11±5.15 Bb | 115.44±1.41 Aa | 372.11±3.00 Aa | 376.19±2.65 Aa | 252.20±23.64 Aa | 253.00±18.33 Aa | 2.00±0.01 Bb | 2.46±0.15 Aa |
| 507 | 127.33±3.06 Aa | 93.78±1.39 Bb | 382.47±0.86 Aa | 378.60±1.08 Bb | 201.63±26.68 Aa | 187.58±19.62 Aa | 3.21±0.04 Aa | 2.33±0.12 Ab |

Note: Data are presented as mean ± SD. Large letter indicates that the difference is extremely significant at the 0.01 level, and small letter indicates that the difference is significant at the 0.05 level.

**Table S4 Changes in osmoregulatory substance content in different lines of spaceflight treatment in *Prunus sibirica* seedlings.*.***

| Line | Soluble sugar content | | Starch content | | Soluble protein content | | Free proline content | |
| --- | --- | --- | --- | --- | --- | --- | --- | --- |
|  | ST | GC | ST | GC | ST | GC | ST | GC |
| 1 | 24.52±1.01 Aa | 20.55±1.01 Ab | 5.96±0.34 Aa | 3.01±0.12 Bb | 2.12±0.10 Aa | 1.43±0.10 Bb | 8.36±0.04 Aa | 7.18±0.08 Bb |
| 28 | 24.40±0.50 Aa | 21.93±0.23 Bb | 4.02±0.05 Aa | 2.54±0.12 Bb | 1.57±0.03 Bb | 1.79±0.05 Aa | 9.32±0.05 Aa | 6.29±0.08 Bb |
| 207 | 27.21±0.54 Aa | 17.51±0.42 Bb | 4.64±0.18 Aa | 3.34±0.05 Bb | 1.72±0.04 Aa | 1.35±0.05 Bb | 9.15±0.07 Aa | 7.13±0.10 Bb |
| 453 | 25.19±0.29 Aa | 20.46±0.70 Bb | 4.06±0.08 Aa | 2.75±0.05 Bb | 1.66±0.06 Aa | 1.76±0.03 Aa | 9.44±0.31 Aa | 6.50±0.13 Bb |
| 507 | 21.78±0.39 Aa | 19.19±0.26 Bb | 4.86±0.09 Aa | 4.24±0.10 Bb | 3.27±0.01 Aa | 2.55±0.08 Bb | 13.12±0.22 Aa | 7.27±0.35 Bb |

Note: Data are presented as mean ± SD. Large letter indicates that the difference is extremely significant at the 0.01 level, and small letter indicates that the difference is significant at the 0.05 level.

**Table S5 Changes in photosynthetic pigment content in different lines of spaceflight treatment in *Prunus sibirica* seedlings.**

| Line | Chl a content | | Chl b content | | Total Chl content | | Car content | |
| --- | --- | --- | --- | --- | --- | --- | --- | --- |
|  | ST | GC | ST | GC | ST | GC | ST | GC |
| 1 | 1.57±0.02 Aa | 1.32±0.02 Bb | 0.63±0.01 Aa | 0.53±0.02 Bb | 2.20±0.02 Aa | 1.85±0.01 Bb | 0.38±0.04 Aa | 0.27±0.02 Aa |
| 28 | 1.66±0.02 Aa | 1.25±0.01 Bb | 0.60±0.01 Aa | 0.54±0.01 Aa | 2.26±0.01 Aa | 1.79±0.02 Bb | 0.48±0.04 Aa | 0.34±0.02 Bb |
| 207 | 1.35±0.05 Aa | 1.21±0.01 Aa | 0.56±0.01Aa | 0.47±0.07 Bb | 1.91±0.07 Aa | 1.69±0.02 Bb | 0.45±0.02 Aa | 0.28±0.02 Bb |
| 453 | 1.43±0.04 Aa | 1.42±0.03 Aa | 0.62±0.02 Aa | 0.60±0.04 Aa | 2.05±0.04 Aa | 2.03±0.01 Aa | 0.52±0.03 Aa | 0.37±0.02 Bb |
| 507 | 1.39±0.01Aa | 1.31±0.01 Bb | 0.65±0.02 Aa | 0.58±0.01 Bb | 2.04±0.01Aa | 1.89±0.02 Bb | 0.40±0.02 Aa | 0.26±0.02 Bb |

Note: Data are presented as mean ± SD. Large letter indicates that the difference is extremely significant at the 0.01 level, and small letter indicates that the difference is significant at the 0.05 level.
