# Supplementary material for: Alterations in physiological and biochemical characteristics of Prunus sibirica seedlings raised from spaceflight seeds
Source: PLoS One. 2025 Apr 24;20(4):e0321147. doi: 10.1371/journal.pone.0321147 (PMC12021159; doi:10.1371/journal.pone.0321147)
Supplement: S3 Table — (DOCX) [file pone.0321147.s003.docx]

**Supporting Information captions**

**S3 Table Changes in antioxidant enzyme activity and MDA levels in different lines of spaceflight treatment *of Prunus sibirica* seedlings*.***

| Line | POD activity | | SOD activity | | CAT activity | | MDA content | |
| --- | --- | --- | --- | --- | --- | --- | --- | --- |
|  | ST | GC | ST | GC | ST | GC | ST | GC |
| 1 | 84.44±2.58 Bb | 122.89±5.04 Aa | 378.39±1.12 Aa | 375.79±1.39 Aa | 302.46±20.42 Aa | 164.58±8.44 Bb | 2.63±0.07 Bb | 2.88±0.05 Aa |
| 28 | 78.67±3.04 Aa | 66.78±3.12 Ab | 380.60±1.07 Aa | 368.36±1.13 Bb | 144.79±13.02 Ab | 201.79±15.77 Aa | 3.15±0.02 Aa | 2.63±0.01 Bb |
| 207 | 155.89±5.94 Aa | 81.44±1.95 Bb | 374.45±2.46 Aa | 360.33±2.77 Bb | 387.83±20.64 Aa | 169.46±22.00 Bb | 3.06±0.18 Aa | 2.34±0.07 Ab |
| 453 | 77.11±5.15 Bb | 115.44±1.41 Aa | 372.11±3.00 Aa | 376.19±2.65 Aa | 252.20±23.64 Aa | 253.00±18.33 Aa | 2.00±0.01 Bb | 2.46±0.15 Aa |
| 507 | 127.33±3.06 Aa | 93.78±1.39 Bb | 382.47±0.86 Aa | 378.60±1.08 Bb | 201.63±26.68 Aa | 187.58±19.62 Aa | 3.21±0.04 Aa | 2.33±0.12 Ab |

Note: Data are presented as mean ± SD. Large letter indicates that the difference is extremely significant at the 0.01 level, and small letter indicates that the difference is significant at the 0.05 level.
