# Supplementary material for: Alterations in physiological and biochemical characteristics of Prunus sibirica seedlings raised from spaceflight seeds
Source: PLoS One. 2025 Apr 24;20(4):e0321147. doi: 10.1371/journal.pone.0321147 (PMC12021159; doi:10.1371/journal.pone.0321147)
Supplement: S4 Table — (DOCX) [file pone.0321147.s004.docx]

**Supporting Information captions**

**S4 Table Changes in osmoregulatory substance content in different lines of spaceflight treatment in *Prunus sibirica* seedlings.*.***

| Line | Soluble sugar content | | Starch content | | Soluble protein content | | Free proline content | |
| --- | --- | --- | --- | --- | --- | --- | --- | --- |
|  | ST | GC | ST | GC | ST | GC | ST | GC |
| 1 | 24.52±1.01 Aa | 20.55±1.01 Ab | 5.96±0.34 Aa | 3.01±0.12 Bb | 2.12±0.10 Aa | 1.43±0.10 Bb | 8.36±0.04 Aa | 7.18±0.08 Bb |
| 28 | 24.40±0.50 Aa | 21.93±0.23 Bb | 4.02±0.05 Aa | 2.54±0.12 Bb | 1.57±0.03 Bb | 1.79±0.05 Aa | 9.32±0.05 Aa | 6.29±0.08 Bb |
| 207 | 27.21±0.54 Aa | 17.51±0.42 Bb | 4.64±0.18 Aa | 3.34±0.05 Bb | 1.72±0.04 Aa | 1.35±0.05 Bb | 9.15±0.07 Aa | 7.13±0.10 Bb |
| 453 | 25.19±0.29 Aa | 20.46±0.70 Bb | 4.06±0.08 Aa | 2.75±0.05 Bb | 1.66±0.06 Aa | 1.76±0.03 Aa | 9.44±0.31 Aa | 6.50±0.13 Bb |
| 507 | 21.78±0.39 Aa | 19.19±0.26 Bb | 4.86±0.09 Aa | 4.24±0.10 Bb | 3.27±0.01 Aa | 2.55±0.08 Bb | 13.12±0.22 Aa | 7.27±0.35 Bb |

Note: Data are presented as mean ± SD. Large letter indicates that the difference is extremely significant at the 0.01 level, and small letter indicates that the difference is significant at the 0.05 level.
