# Supplementary material for: Alterations in physiological and biochemical characteristics of Prunus sibirica seedlings raised from spaceflight seeds
Source: PLoS One. 2025 Apr 24;20(4):e0321147. doi: 10.1371/journal.pone.0321147 (PMC12021159; doi:10.1371/journal.pone.0321147)
Supplement: S5 Table — (DOCX) [file pone.0321147.s005.docx]

**Supporting Information captions**

**Table S5 Changes in photosynthetic pigment content in different lines of spaceflight treatment in *Prunus sibirica* seedlings.**

| Line | Chl a content | | Chl b content | | Total Chl content | | Car content | |
| --- | --- | --- | --- | --- | --- | --- | --- | --- |
|  | ST | GC | ST | GC | ST | GC | ST | GC |
| 1 | 1.57±0.02 Aa | 1.32±0.02 Bb | 0.63±0.01 Aa | 0.53±0.02 Bb | 2.20±0.02 Aa | 1.85±0.01 Bb | 0.38±0.04 Aa | 0.27±0.02 Aa |
| 28 | 1.66±0.02 Aa | 1.25±0.01 Bb | 0.60±0.01 Aa | 0.54±0.01 Aa | 2.26±0.01 Aa | 1.79±0.02 Bb | 0.48±0.04 Aa | 0.34±0.02 Bb |
| 207 | 1.35±0.05 Aa | 1.21±0.01 Aa | 0.56±0.01Aa | 0.47±0.07 Bb | 1.91±0.07 Aa | 1.69±0.02 Bb | 0.45±0.02 Aa | 0.28±0.02 Bb |
| 453 | 1.43±0.04 Aa | 1.42±0.03 Aa | 0.62±0.02 Aa | 0.60±0.04 Aa | 2.05±0.04 Aa | 2.03±0.01 Aa | 0.52±0.03 Aa | 0.37±0.02 Bb |
| 507 | 1.39±0.01Aa | 1.31±0.01 Bb | 0.65±0.02 Aa | 0.58±0.01 Bb | 2.04±0.01Aa | 1.89±0.02 Bb | 0.40±0.02 Aa | 0.26±0.02 Bb |

Note: Data are presented as mean ± SD. Large letter indicates that the difference is extremely significant at the 0.01 level, and small letter indicates that the difference is significant at the 0.05 level.
